# Co-Induction of a Glutathione-*S*-transferase, a Glutathione Transporter and an ABC Transporter in Maize by Xenobiotics

**DOI:** 10.1371/journal.pone.0040712

**Published:** 2012-07-11

**Authors:** Sen Pang, Liusheng Duan, Zhiqian Liu, Xiaoyu Song, Xuefeng Li, Chengju Wang

**Affiliations:** 1 Engineering Research Center of Plant Growth Regulators, Ministry of Education, College of Agronomy and Biotechnology, China Agricultural University, Beijing, People’s Republic of China; 2 College of Sciences, China Agricultural University, Beijing, People’s Republic of China; 3 Department of Primary Industries, Biosciences Research Division, Victorian AgriBiosciences Centre, Bundoora, Victoria, Australia; University of South Florida College of Medicine, United States of America

## Abstract

Glutathione conjugation reactions are one of the principal mechanisms that plants utilize to detoxify xenobiotics. The induction by four herbicides (2,4-D, atrazine, metolachlor and primisulfuron) and a herbicide safener (dichlormid) on the expression of three genes, *ZmGST27*, *ZmGT1* and *ZmMRP1*, encoding respectively a glutathione-*S*-transferase, a glutathione transporter and an ATP-binding cassette (ABC) transporter was studied in maize. The results demonstrate that the inducing effect on gene expression varies with both chemicals and genes. The expression of *ZmGST27* and *ZmMRP1* was up-regulated by all five compounds, whereas that of *ZmGT1* was increased by atrazine, metolachlor, primisulfuron and dichlormid, but not by 2,4-D. For all chemicals, the inducing effect was first detected on *ZmGST27.* The finding that *ZmGT1* is activated alongside *ZmGST27* and *ZmMRP1* suggests that glutathione transporters are an important component in the xenobiotic detoxification system of plants.

## Introduction

Metabolism of herbicides in plants can generally be divided into three phases. In phase I, the herbicide may be oxidized, reduced or hydrolyzed to introduce or reveal a functional group. In phase II, the herbicide is conjugated to glutathione, glucose or malonate by the respective transferase to form a water-soluble conjugate. In phase III, herbicide conjugates are transported from the cytosol to the vacuole for further degradation [1].

Glutathione-*S*-transferases (GSTs, E.C.2.5.1.18) are a large family of enzymes that catalyze the conjugation of the reduced glutathione (GSH) to a variety of electrophilic xenobiotics to yield less- or non-toxic derivatives [2], [3]. GSTs have been found in virtually all plants, vertebrates, insects, yeasts, and bacteria. Plant GSTs have been extensively studied because of their ability to detoxify herbicides [4], [5]. GSTs are also implicated in the herbicide resistance of weeds; and the correlation between herbicide resistance and increased GST activity was established in some weed species [6], [7]. In addition, herbicide safeners, chemicals that protect crop plants from herbicide injury, are also able to enhance the expression of GSTs in various plants [8], [9].

Increases in GSH and GSTs levels have been linked to plant resistance and adaptation to a variety of physical and chemical stresses encountered in the environment [10]. However, accumulation of the conjugated products in cells can lead to a decrease in the detoxification activity of the phase II system. Several glutathione conjugates (GS conjugates) have been found to inhibit both glutathione-*S*-transferase and glutathione reductase activities [11]. It is generally believed that the various GS conjugates are transported into the vacuole by tonoplast transporters for further degradation [12], [13].

ATP binding cassette (ABC) transporters were reported to transport glutathione conjugates [12]. Multidrug resistance-associated proteins (MRPs), members of ABC transporter family, play a role in the detoxification of xenobiotics by transporting GS conjugates into the vacuole [12], [13]. Several studies have shown that MRPs of plants are inducible by herbicide safeners [14], [15]. Surprisingly, few reports can be found concerning the effect of herbicides on the expression of MRPs.

In addition to ABC transporters located in the tonoplast, recent studies showed that glutathione transporters, located in the plasma membrane, were able to mediate the transport of both GSH and GS conjugates [16], [17]. Our earlier work found that the expression of a glutathione transporter gene isolated from maize, named *ZmGT1,* was inducible by herbicides atrazine and metolachlor and the inducing effect of metolachlor in different maize cultivars was correlated to their tolerance to this herbicide, suggesting an involvement of *ZmGT1* in the detoxification of xenobiotics by plants [18], [19].

It is generally established that the detoxification of herbicides in plants is a multi-step process, requiring the participation of GSH, detoxifying enzymes such as GSTs, cytochrome P450 monooxygenases (P450) and glycosyltransferases, and ABC transporters [15], [20], [21]. However, evidence supporting this statement comes mostly from isolated studies using different plant materials and chemicals, and dealing with one factor in each study. Theodoulou et al reported the co-induction of GSTs and MRPs in wheat, but only one herbicide safener was tested [14]. Our initial work tested the effect of three herbicides (metolachlor, atrazine and 2,4-D) on the expression of *ZmGT1*s in maize, but no parallel analysis was conducted on other detoxifying genes [19]. In this study, we have compared the expression pattern of a glutathione-*S*-transferase (*ZmGST27*), an ABC transporter (*ZmMRP1*) and a glutathione transporter (*ZmGT1*) in maize leaves after treatment by a range of herbicides and a herbicide safener. The co-induction of the three genes by most chemicals tested strongly suggests that glutathione transporters are also one of the components in the xenobiotic detoxification system of plants.

## Results

### Induction of *ZmGST27* Expression by Chemicals

**Figure 1:**
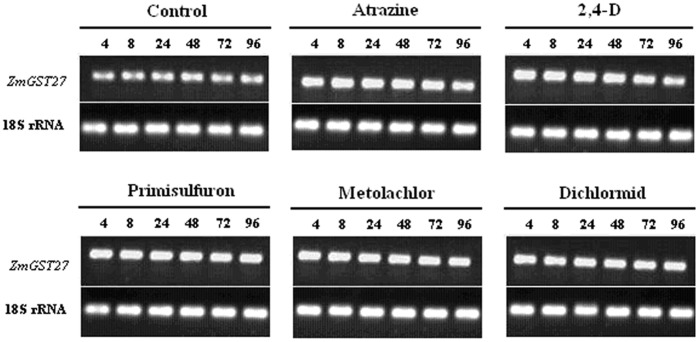
Semi-quantitative RT-PCR analysis of *ZmGST27* expression in maize leaves after treatment by various chemicals. The numbers (4, 8, 24, 48, 72 and 96) above each lane indicate the time intervals (h) after treatment. The experiment was repeated twice with similar results.

A time course study was designed to compare the expression of *ZmGST27* in maize leaves at 4, 8, 24, 48, 72 and 96 h after treatment by atrazine, 2,4-D, primisulfuron, metolachlor or dichlormid. The semi-quantitative RT-PCR results showed that the expression of *ZmGST27* was induced by all five test compounds. The inducing effect on gene expression was observable at 4 h after treatment and maintained for the entire duration of the experiment (96 h after treatment). No significant difference was found between different chemicals in their ability to increase the transcript level of *ZmGST27* (Fig. 1).

### Induction of *ZmMRP1* by Chemicals

**Figure 2:**
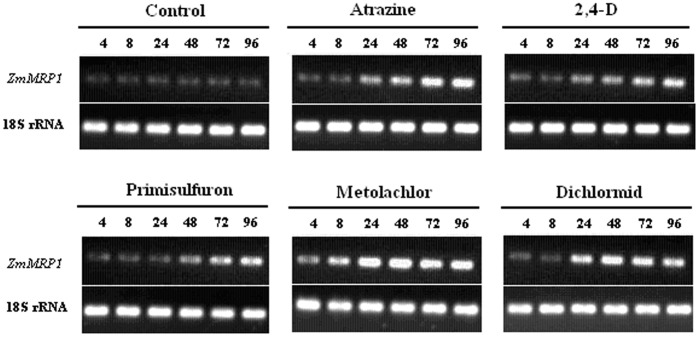
Semi-quantitative RT-PCR analysis of *ZmMRP1* expression in maize leaves after treatment by various chemicals. The numbers (4, 8, 24, 48, 72 and 96) above each lane indicate the time intervals (h) after treatment. The experiment was repeated twice with similar results.

Semi-quantitative RT-PCR was also used to evaluate the influence of the five compounds on the expression of *ZmMRP1* in maize leaves. All five compounds significantly increased the amount of *ZmMRP1* transcript, but at different magnitude and after different time delay. The greatest promoting effect was observed with metolachlor, followed by dichlormid and atrazine, and then 2,4-D and primisulfuron. While the inducing effect could be detected at 4 h after treatment in the case of metolochlor, significant up-regulation on gene expression was observed only after 24 h or 48 h treatment for the other chemicals (Fig. 2).

### Induction of *ZmGT1* by Chemicals

**Figure 3:**
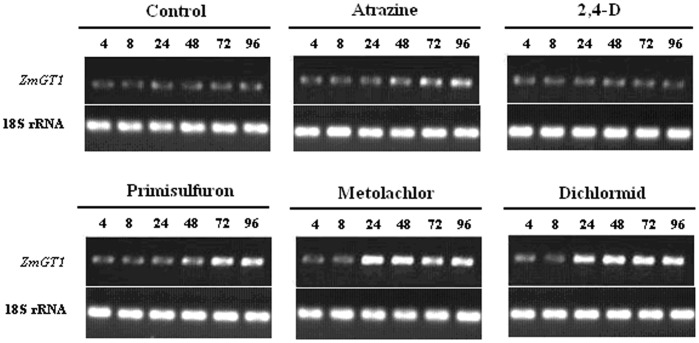
Semi-quantitative RT-PCR analysis of *ZmGT1* expression in maize leaves after treatment by various chemicals. The numbers (4, 8, 24, 48, 72 and 96) above each lane indicate the time intervals (h) after treatment. The experiment was repeated twice with similar results.

The effects of the five chemicals on the expression of *ZmGT1* in maize leaves were also compared at 4, 8, 24, 48, 72 and 96 h after treatment. While 2,4-D treatment had no influence on *ZmGT1* expression, the other four compounds exhibited a significant inducing effect. Metolachlor and dichlormid appear to be the strongest inducer for *ZmGT1* expression, followed by primisulfuron, and then atrazine. The inducing effect became detectable after 24 h (for metolachlor and dichlormid) or 48 h (for primisulfuron and atrazine) treatments (Fig. 3).

## Discussion

The role of GSTs in the detoxification of certain herbicides such as alachlor, metolachlor and atrazine, has been known for many years. This study shows that the expression of *ZmGST27* can be induced by all tested compounds including 2,4-D and primisulfuron, two herbicides not known to be metabolized through glutathione conjugation. The up-regulation of *ZmGST27* expression by these last two compounds is likely to be a general response of this gene to chemical stress. More compounds of different structures should be tested to ascertain this. The activation of GST genes in response to biotic and abiotic stresses has been reported previously [22], [23].

Plant ABC transporters belonging to the MRPs subfamily were shown to be able to transport GS conjugates and their role in herbicide metabolism has been well established [20]. It was also reported that ABC transporters play a role in vacuolar sequestration of the glucose conjugates of certain herbicides and plant hormones [12], [24]. This could explain why the *ZmMRP1* expression was also inducible by 2,4-D and primisulfuron, two herbicides known to be metabolized through glycosylation [17], [25].

Our earlier work has provided evidence for the involvement of a glutathione transporter, *ZmGT1,* in the detoxification of atrazine and metolachlor in maize [18], [19]. However, a comprehensive analysis of the expression patterns of GSTs, ABC transporters and glutathione transporters in the same plant tissue after exposure to the same chemicals was lacking. This work, through comparing the expression of the three types of genes after treatment by a range of chemicals, provides new insights into the plant detoxification system and reveals that glutathione transporters are activated alongside GSTs and ABC transporters upon the invasion of toxic compounds. How the function of these two types of transporters is coordinated temporally and spatially remains to be elucidated.

The lack of effect of 2,4-D on the expression of *ZmGT1* appears to suggest that glutathione transporters are unable to transport glucose conjugates and thus have a narrower spectrum of substrates as compared to ABC transporters. Again more compounds should be tested to confirm this. The finding that *ZmGT1* is inducible by primisulfuron is unexpected since this herbicide is known to be metabolized through glycosylation. However, Cagnac et al also found that a glutathione transporter from Arabidopsis (AtOPT6) was induced by the same herbicide [17]. It is possible that glutathione transporters are involved in other pathways of the detoxification process.

Herbicide safeners are known to increase the activity of GSTs, thus accelerating the metabolism of themselves and that of herbicides [26], [27]. Dichlormid, a herbicide safener of maize, has been reported to increase the levels of *ZmGST27* and *ZmMRP1* transcripts [28], [29]. In this work we show that this compound also increases the amount of *ZmGT1* transcript. This further suggests that glutathione transporters participate in the multi-step xenobiotic detoxification process.

In summary, simultaneous analysis of the expression of *ZmGST27*, *ZmMRP1* and *ZmGT1* after treatment by a range of chemicals allows us to demonstrate that, in addition to GSH, GSTs and ABC transporters, glutathione transporters located in the plasma membrane are also an important component in the glutathione conjugation-related plant detoxification system. This study also shows that besides the glutathione conjugation-related process, GSTs and ABC transporters may be involved in other metabolic pathways in response to chemical stress.

## Materials and Methods

### Herbicides

Atrazine (2-chloro-4-(ethylamino)-6-(isopropylamino)-s-triazine, 97% purity), 2,4-D (2,4-dichlorophenoxyacetic acid, 96% purity), primisulfuron (2-[4,6-bis(difluoromethoxy)pyrimidin-2-ylcarbamoylsulfamoyl]benzoic acid, 95% purity), metolachlor 2-chloro-N-(2-ethyl-6-methylphenyl)-N-(2-methoxy-1-methylethyl) acetamide, 95% purity) and dichlormid (N, N-diallyl-2, 2-dichloroacetamide, 97% purity) were kind gifts from Institute of Plant Protection, Chinese Academy of Agricultural Sciences.

### Plant Material

Maize (*Zea mays*, cv Bainuo No. 2) were grown on sand in the glasshouse as described previously [18]. Plants grown to the 3-leaf stage were transferred to a hydroponic culture (three plants per pot). Each pot contained 50 mL of nutrient solution. Plants were adapted for 7 d and the nutrient solution was changed every 2 d. After the 7 d adaptation period, the herbicides or safener were added into the nutrient solution to a final concentration of 100 µM and the nutrient solution was changed daily. At the end of the treatment, leaves were harvested, immediately frozen in liquid nitrogen, and stored at −80°C.

### RNA Isolation

Frozen leaf samples were ground into a fine powder in liquid nitrogen. Total RNA was isolated using TRIzol reagent (Invitrogen, USA), and then digested by DNase I (TakaRa, China). First strand cDNA was synthesized with AMV reverse transcriptase and Oligo(dT)_15_, according to the manufacturer’s protocol of Reverse Transcription System (Promega, USA).

### Semi-quantitative RT-PCR Analysis of Transcripts

Three primer pairs (*ZmGST27-F* 5′- GAC CTG CTC CTC GCC TCC AA -3′and *ZmGST27-R* 5′- CCT CCA GCG TGT CCA TAG CG -3′; *ZmGT1-F* 5′- GTG CCG CAG TGG TGG TTC -3′ and *ZmGT1* 5′- GTG ACG ACG AAG GCG AGC -3′; *ZmMRP1-F* 5′- CTA GAA TAT GAA ACA CCA GCC AAG -3′and *ZmMRP1-R* 5′- CTG CAA TAA TGG TAG ATC ATG TTG -3′) were designed from the ORF region of *ZmGST27* (accession number AF244692), *ZmGT1* (accession number FJ573212), and *ZmMRP1* (accession number AY186244) respectively to amplify a single fragment for each gene. Another pair of primers (P3 5′- GCT CTT TCT TGA TTC TAT GGG TGG -3′ and P4 5′- GTT AGC AGG CTG AGG TCT CGT TC -3′) was used to amplify a fragment from maize 18S ribosomal RNA (accession number M82386), chosen as an internal reference. PCR amplification conditions were: 30 s at 95°C, 30 s at 62°C, 30 s at 72°C (30 cycles).
